# Early severe HIV disease precedes early antiretroviral therapy in infants: Are we too late?

**DOI:** 10.7448/IAS.17.1.18914

**Published:** 2014-06-11

**Authors:** Steve Innes, Erica Lazarus, Kennedy Otwombe, Afaaf Liberty, Ramona Germanus, Anita Janse Van Rensburg, Nelis Grobbelaar, Theunis Hurter, Brian Eley, Avy Violari, Mark F Cotton

**Affiliations:** 1 Children’s Infectious Diseases Clinical Research Unit (KID-CRU), Stellenbosch University and Tygerberg Children’s Hospital, Cape Town, South Africa; 2 Perinatal HIV Research Unit, Faculty of Health Sciences, University of the Witwatersrand, Johannesburg and Chris Hani Baragwanath Hospital, Soweto, South Africa; 3 Anova Health Institute, Paarl, Western Cape, South Africa; 4 Red Cross War Memorial Children’s Hospital and the University of Cape Town, Cape Town, South Africa

**Keywords:** infant HIV, antiretroviral therapy, South Africa, early infant diagnosis, programmatic cohort

## Abstract

**Objective:**

To describe the degree of HIV disease progression in infants initiating antiretroviral therapy (ART) by three months of age in a programmatic setting in South Africa.

**Design:**

This was a programmatic cohort study.

**Methods:**

Electronic and manual data extraction from databases and antiretroviral registers in 20 public clinics in Cape Town and electronic data extraction from a large ART service at Chris Hani Baragwanath Hospital in Soweto were performed. Records of all infants initiated on ART by three months of age between June 2007 and September 2010 were extracted. Demographics, immunological and clinical stage at ART initiation were analyzed descriptively by chi-square, two-sample *t*-test and Kaplan–Meier methods.

**Results:**

A total of 403 records were identified: 88 in Cape Town and 315 in Soweto. Median age at ART initiation was 8.4 [interquartile range (IQR): 7.2–9.7] weeks. At ART initiation, 250 infants (62%) had advanced HIV disease (CD4% <25% or absolute CD4<1500 cells/mm^3^ or WHO clinical Stage 3 or 4). Median age at ART initiation by site was 10.3 (IQR: 8.2–11.9) weeks in Cape Town and 8.6 (IQR: 7.7–10.0) weeks in Soweto infants (*p*<0.0001). In Cape Town, 73 infants (83%) had advanced HIV disease at ART initiation, compared to 177 infants (56%) in Soweto (*p*<0.0001). On logistic regression, each month increase in age at ART initiation lowered the odds of initiating ART in an optimal state (OR: 0.56, CI: 0.36–0.94) and increased the odds of advanced HIV disease at ART initiation (OR: 1.69, CI: 1.05–2.71).

**Conclusions:**

ART initiation by three months of age may not adequately prevent disease progression. New emphasis on early diagnosis and rapid initiation of ART in the first weeks of life are essential to further reduce infant mortality.

## Introduction

Despite a 43% reduction in mother-to-child transmission (MTCT) of HIV, 390,000 children globally were newly infected with the virus in 2010 [1]. Of these, 48,000 occurred in South Africa where the perinatal transmission of HIV was 3.5% [2]. Pooled national data showed that only 54% of HIV-exposed infants received a HIV DNA polymerase chain reaction (PCR) test by two months of age in 2010 [3]. Delayed diagnosis leads to delay in access to antiretroviral therapy (ART) and subsequent increase in infant morbidity and mortality [4, 5]. The Children with HIV Early antiRetroviral therapy (CHER) trial showed that ART initiation before 12 weeks of age in infants with CD4≥25% and minimal clinical HIV disease reduced mortality by 75% [4]. Worryingly, however, 19% of the infants screened for CHER (median age: seven weeks) already had severe CD4 depletion (CD4<25%). A retrospective review of the South African national infant death register data demonstrated a peak in early infant mortality due to HIV between two and three months of age [6]. Similarly, a pooled analysis of sub-Saharan African data revealed substantial mortality within the first 3 months of life in perinatally infected infants [7]. These data imply that even when early infant diagnosis and referral occur, ART initiation by three months of age may be too late for a significant proportion of HIV-infected infants. The current study compares the pre-ART characteristics of infants who were successfully initiated on ART by three months of age in Cape Town and Soweto, and highlights the proportion who had advanced HIV disease at ART initiation.

## Method

In Cape Town, HIV-exposed infants born at Midwife Obstetric Units were routinely referred to their local clinic for infant PCR testing at four to six weeks of age; results were given two to four weeks later and those who were infected were referred on to a paediatric ART treatment site to initiate ART. In Soweto (which, as a whole, forms Johannesburg Health Subdistrict D), prior to 2010, any infant newly diagnosed with HIV was routinely referred to Chris Hani Baragwanath Academic Hospital (CHBAH) to initiate ART, since staff at other health facilities were not trained to initiate infant ART prior to the introduction of the paediatric nurse-initiated ART training programme in 2010. At CHBAH, HIV-positive mothers were routinely given a referral at neonatal discharge to return for PCR testing on-site at the Paediatric Wellness Clinic at four weeks of age. At the follow-up visit two weeks later, test results were given and ART initiation processes were initiated on-site. Infants referred for ART from outside CHBAH would routinely be seen at the Paediatric Wellness Clinic, and therefore the cases on the Paediatric Wellness database would have included, as far as possible, all newly diagnosed infants in Soweto. Acutely ill children who were diagnosed with HIV during inpatient admission at CHBAH would not typically appear on the Paediatric Wellness database until after discharge, since they were usually initiated on ART during their inpatient stay.

Records of all infants initiated on ART by three months of age between June 2007 and September 2010 were extracted. Infants who died or were lost to follow-up before ART could be initiated, or who were initiated on ART after three months of age, were not included. In Cape Town, all primary, secondary and tertiary public health care facilities providing paediatric ART in the Northern, Eastern, Khayelitsha, Tygerberg, Drakenstein and Stellenbosch health subdistricts were included, along with the Red Cross War Memorial Children’s Hospital (RXH) in the Western subdistrict. Electronic data extraction from the Tygerberg Family Clinic for HIV database, the Red Cross Paediatric HIV database and the Winelands Paediatric HIV database was performed, with manual data extraction from antiretroviral registers in the remaining clinics. In Soweto, electronic data extraction was from the Paediatric Wellness Clinic database at CHBAH, which is run and maintained by the Perinatal HIV Research Unit. This database used a data fax system to collect data in real time at each infant visit. Infants were not primarily research participants.

Demographic characteristics, pre-ART immunological and clinical stage were recorded. Mortality and loss-to-follow-up (LTFU) status at date of data extraction were also recorded. Participants were described as being lost to follow-up if they had not been seen in the clinic for more than a year. Advanced HIV disease was defined as WHO clinical stages 3 or 4 or immunodeficiency (CD4% <25% or absolute CD4<1500 cells/mm^3^) as per WHO guidelines [8]. Descriptive statistics was performed on continuous variables while chi-square analysis was used for categorical comparisons. The pre-ART immunological values were compared using the two-sample *t*-test. Time-to-event analysis was performed using Kaplan–Meier methods in which comparisons were made using the log-rank test. Logistic regression was used to determine the predictive factors for initiating ART in an optimal clinical and immunological state (defined as WHO clinical Stage 1 or 2 and normal CD4) and for advanced HIV disease at ART initiation. Normal CD4 was defined as CD4% ≥25% and absolute CD4≥1500 cells/mm^3^. Statistical analyses were performed using R version 2.10.0 (Bell Laboratories, New Jersey). Stata version 11.0 (Statacorp, Texas) was used for survival analysis and MedCalc version 12.2.1 for graphics. Where data were missing, these individuals were included in the denominator when percentage was calculated, unless stated otherwise. This study was approved by the Health Research Ethics Committees of Stellenbosch University and the Universities of the Witwatersrand and Cape Town, and was performed in accordance with the Declaration of Helsinki (version 2000).

## Results

Eighty-eight records were identified in Cape Town and 315 in Soweto (Table 1). Median age at ART initiation was 8.4 [interquartile range (IQR): 7.2–9.7] weeks. By ART initiation before three months of age, 250 (62%) infants had advanced HIV disease (as defined earlier): 73 (83%) in Cape Town and 177 (56%) in Soweto (*p*<0.0001). Median (IQR) CD4% and absolute CD4 amongst the 95 infants with WHO clinical Stage 3 or 4 at ART initiation was 28% (20–33%) and 1318 (740–1789) cells/mm^3^, respectively, of whom 56 (59%) had CD4% <25% or absolute CD4<1500 cells/mm^3^. Only 22 (5%) infants were initiated on ART before six weeks of age, of whom six were initiated on ART in an optimal state (as defined earlier) and nine had advanced HIV disease at ART initiation. The number of infants initiating ART before six weeks of age was too small to allow meaningful stratification. There was no significant gender difference among those with advanced HIV disease or among those initiating ART in an optimal state (*p*=0.11 for both).

**Table 1:** Characteristics of infants who were successfully initiated on antiretroviral therapy (ART) by three months of age in the two centres

	Total, *N*=403	Cape Town, *N*=88	Soweto, *N*=315	p*
Demographics				
Median age at ART initiation in weeks (IQR)	8.4 (7.2–9.7)	10.3 (8.2–11.9)	8.6 (7.7–10.0)	<0.0001
Gender (male/female)	170/209	41/40	129/169	0.24
Pre-ART CD4				
Median pre-ART CD4% (IQR)	29% (23–37%)	27% (18–35%)	28% (22–34%)	0.55
Median pre-ART absolute CD4 in cells/mm^3^ (IQR)	1535 (1056–2247)	1387 (717–2188)	1513 (1059–2185)	0.20
Number with abnormal pre-ART CD4 (CD4% <25% or absolute CD4 <1500 cells/mm^3^)	207 (51%)	44 (50%)	163 (52%)	0.77
Missing both CD4% and absolute CD4 data	49 (12%)	4 (5%)	45 (14%)	–
WHO at ART initiation				
WHO Stage 1	209 (63%)	8 (14%)	201 (74%)	<0.0001
WHO Stage 2	25 (8%)	9 (15%)	16 (6%)	
WHO Stage 3	72 (22%)	23 (39%)	49 (18%)	
WHO Stage 4	23 (7%)	19 (32%)	4 (2%)	
Missing WHO clinical stage data	74	29	45	–
Summary condition at ART initiation				
Number initiating in an “optimal” state (Normal CD4 and WHO 1 or 2)#	106 (26%)	13 (15%)	93 (30%)	0.005
Number missing either CD4 (CD4% or absolute CD4) or WHO stage data#	79 (20%)	33 (38%)	46 (15%)	–
Number with advanced HIV disease (pre-ART CD4% <25% or absolute CD4 <1500 cells/mm^3^ or WHO Stage 3 or 4)	250 (62%)	73 (83%)	177 (56%)	<0.0001
Number missing both CD4 (CD4% and absolute CD4) and WHO stage data	44 (11%)	0 (0%)	44 (14%)	–

* Comparing Cape Town to Soweto;

# children were included if either CD4% or absolute CD4 was available. Where both were available, both were required to be normal; SD=standard deviation; WHO=World Health Organization; IQR=Interquartile range; ART=Antiretroviral therapy.

**Figure 1:**
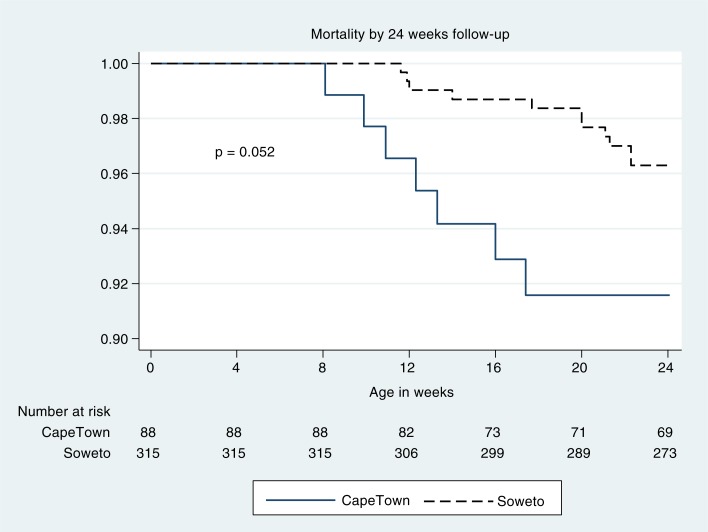
Mortality by 24 weeks of age in infants who were successfully initiated on ART before three months of age.* *Infants who died or were lost to follow-up before ART could be initiated, or who were initiated on ART after three months of age were not included.

**Figure 2:**
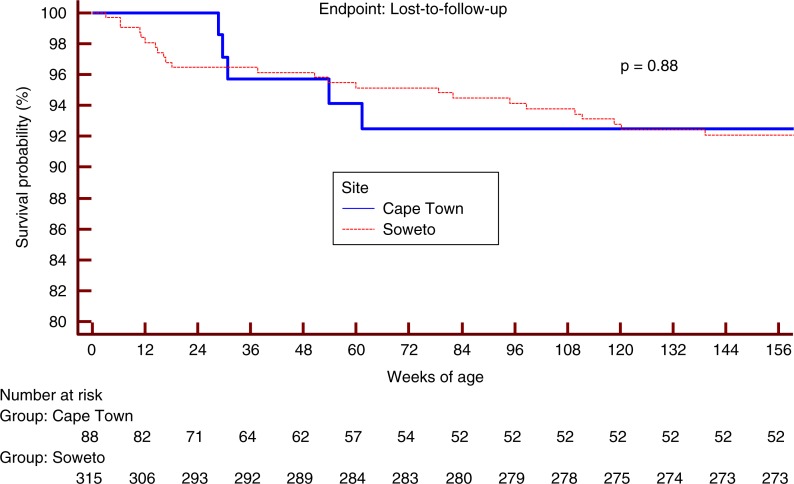
Loss-to-follow-up at the date of data extraction.* *Infants who died or were lost to follow-up before ART could be initiated, or who were initiated on ART after three months of age were not included.

Median age at ART initiation differed by two weeks with the Cape Town infants initiated later than the Soweto infants (*p*<0.0001). ART regimens were similar at all sites and typically consisted of stavudine or zidovudine, lamivudine and lopinavir/ritonavir. Mortality and LTFU outcomes were available for 653 patient-years of follow-up after ART initiation (median: 1.6; IQR: 0.9–2.4 years of follow-up per patient). Mortality difference by site was marginally significant (*p*=0.05; Figure 1). LTFU was not significantly different at the two sites (*p*=0.88; Figure 2). On logistic regression, each month increase in age at ART initiation lowered the odds of initiating ART in an optimal state (OR: 0.56, CI: 0.36–0.94) and increased the odds of advanced HIV disease at ART initiation (OR: 1.69, CI: 1.05–2.71).

The Cape Town group included 14 infants from RXH, a tertiary hospital in the Western health subdistrict, of whom 10 were WHO clinical Stage 3 and the remainder were WHO clinical Stage 1 or 2. All 14 initiated on ART after six weeks of age with CD4% ≥25%, although nine had an absolute CD4<1500 cells/mm^3^ with a median (IQR) of 1390 (906–1825) cells/mm^3^. These 14 infants are mentioned separately because data from the non-tertiary clinics in that health subdistrict were not available. In the Soweto group, more than 95% of infants were diagnosed on-site at CHBAH, with 16 additional infants referred from local Soweto clinics to initiate ART as outpatients.

In Soweto, over 90% of mother–infant pairs received some form of antiretroviral prevention of mother-to-child transmission (PMTCT): 264 infants received PMTCT (252 received single-dose nevirapine; 176 received post-partum zidovudine); 13 infants received no PMTCT (although 8 out of 13 of those mothers did receive PMTCT, which consisted of single-dose nevirapine for six mothers; post-partum zidovudine for one mother; and three-drug ART for two mothers); and 35 infants had no PMTCT status recorded (although 16 out of 35 of those mothers did receive PMTCT, which consisted of single-dose nevirapine for 16 mothers and post-partum zidovudine for two mothers). In Cape Town, PMTCT data were not available for the majority of infants.

Median (IQR) baseline HIV RNA viral load among Cape Town infants was 365,000 copies/ml (76,000–1,800,000 copies/ml), with 36 infants’ data missing. In Soweto, baseline viral load data was not routinely collected.

## Discussion

The current study demonstrates that ART initiation between 8 and 12 weeks of age is associated with advanced HIV disease at ART initiation. Previous studies have shown that ART initiation at an advanced stage of HIV disease is associated with poorer clinical and immune recovery [9, 10]. In the PMTCT programmes of the highest burden countries in sub-Saharan Africa, a PCR test is performed at six weeks of age in HIV-exposed infants, followed ideally by HIV PCR results by 10 weeks of age. If HIV-infected, then pre-treatment counselling is given with the aim of initiating ART by three months of age [11]. The results of the current study, however, suggest that ART initiation by three months of age may be too late for over half the infants who reach this age. In addition, recent data have suggested that delaying infant testing until six weeks of age may lead to significant delays in ART initiation, high LTFU rates and a significant number of missed diagnoses [12].

Although more than half of the infants in this study had advanced disease at ART initiation, there was a noticeable difference between the two sites. This may be partially explained by differences in PMTCT programmes between the provinces: the rollout of triple ART from 14 weeks of gestation for women with CD4<200 cells/mm^3^ began in 2004, with a single dose of nevirapine in labour for those with CD4>200 cells/mm^3^. From February 2008 to July 2010, the South African Department of Health PMTCT guidelines also provided zidovudine monotherapy from 28 weeks of gestation for pregnant women with CD4>200 cells/mm^3^ [13]. In the Western Cape, however, zidovudine monotherapy from 28 weeks of gestation was available from 2006, two years earlier than in Soweto. This broader coverage resulted in fewer transmissions in the Western Cape [3], which was also influenced by the lower antenatal HIV prevalence in the Western Cape province compared to Gauteng province (15% vs. 30%) [14]. Infants infected despite zidovudine PMTCT have rapid progression of HIV if not treated early with ART [15]. Infections occurring despite peripartum PMTCT may have occurred *in utero*. Mortality from *in utero* infection is more rapid than peripartum infection [16], and this may explain part of the difference in degree of disease progression and corresponding mortality rate by site seen in our study. Despite similar guidelines for the timing of infant PCR testing in the Western Cape and Gauteng, HIV-infected infants in the current study started ART significantly later in Cape Town than in Soweto. This relates to an additional visit at four weeks post-partum at CHBAH, which was not routinely offered at the Cape Town sites. While causality is difficult to assign, this delay was associated with more advanced HIV disease at ART initiation. The delay was most likely due to the fragmented distribution of services in Cape Town, where services are spread across multiple sites and mothers must navigate a series of referrals to access ART for their infants. For example, a mother may attend an antenatal clinic at one site; be referred to another site to deliver; be referred to a third site for sixth- and tenth-week infant check-ups; and then, if the infant is HIV-infected, be referred to a fourth site for her infant to initiate ART. Integration of perinatal, infant PCR testing and paediatric ART treatment services may reduce delays in ART initiation for HIV-infected infants and LTFU and reduce the proportion of infants with advanced HIV disease at ART initiation.

It is important to advocate for programmatic changes to achieve early infant diagnosis and rapid initiation of ART to further reduce HIV-related infant mortality and morbidity. The addition of a PCR test at birth to national PMTCT guidelines would identify *in-utero*-infected infants, who are at greatest risk of rapid HIV disease progression [16]. Birth HIV PCR testing has been associated with lower LTFU and mortality at three months of age in infants infected *in utero* [17]. Infants of mothers who did not access adequate antenatal PMTCT (and may thus have high viral loads) are particularly at an increased risk of HIV transmission and rapid disease progression [18]. These “high-risk” infants would benefit most from selective birth PCR testing and, where PMTCT programme resources are limited, policy-makers should consider providing a birth PCR test to infants of mothers who did not access adequate PMTCT. For these selected infants, the PCR result could be routinely followed-up at the one-week obstetric follow-up visit. Infants found to be HIV-infected could be immediately referred for ART initiation. The absolute number of positive birth PCR tests is likely to be limited, making active tracing by the receiving paediatric ART clinic feasible.

## Limitations

Being a retrospective study, the possibility of confounders cannot be excluded. In particular, selection bias is possible since our study was unable to record the infants who died or were lost to follow-up before ART could be initiated, and did not include infants who were initiated on ART after three months of age. The age at PCR testing was not known, and neither the length of delay between testing and ART initiation nor the cause of delay was known. While every effort was made to include all available data sources, the health districts were selected in a non-random manner and thus may not accurately represent the national population. In addition, HIV PCR testing rates were low (36.6% in 2008; 51.8% in 2009 and 59.8% in 2010) [19], and it is possible that a more complete testing rate may have led to a different result. After the study, the authors became aware of 30 infants in Soweto and four infants in Cape Town who did not appear on the respective databases as they were recruited onto the CHER trial. These infants were all WHO clinical Stage 1 or 2 and had CD4% >25% when they were initiated on ART before three months of age; thus, their inclusion would have further increased the differences between sites and would have marginally reduced the overall proportion of infants who had advanced HIV disease at ART initiation. Some data were missing due to deficiencies in routine programmatic data collection and these may have changed the findings. The proportion with missing WHO data is high, and it is possible that those with missing data may be a biased subgroup. Excluding them from the denominator may have distorted the calculated percentages, resulting in over-reporting and increasing the risk of a type 1 error. However, if those with missing data are added to the denominator, the percentage of infants with WHO clinical Stage 3 or 4 disease remains significantly higher in Cape Town than Soweto (48% vs. 17%), and remains high overall (24%). Data from both cities included all new infant HIV diagnoses at primary, secondary and tertiary health care facilities in the respective health subdistricts, minimizing the likelihood of selection bias as far as possible. The exception was the Western health subdistrict of Cape Town for which only the RXH database was included as data from the non-tertiary clinics in that health subdistrict were not available. However, despite being a tertiary hospital, the RXH infants were not more sick and, in fact, had a lower proportion of advanced HIV disease when compared to Cape Town as a whole (71% vs. 76%) and a higher proportion initiated in optimal state (28% vs. 18%). Multiple differences exist between the two study settings and it may be incorrect to ascribe the difference in HIV disease stage to the difference in age at ART initiation. The referral patterns and efficiency of the Cape Town HIV service may have improved since September 2010, and the differences in mortality and outcome may have subsequently lessened. The infants who were initiated on ART within 24 weeks of the date of data extraction would have contributed less follow-up time to the Kaplan–Meier analysis, and the mortality patterns of these children may be significantly different to the rest of the group. Differences in baseline HIV RNA viral load between sites may have led to the difference in HIV disease state; however, viral load data was extremely sparse because, at the time, viral load was not the standard of care.

## Conclusions

ART initiation by three months of age may be too late to adequately prevent HIV disease progression. More than half the infants had advanced HIV disease at the time of ART initiation despite initiating ART by three months of age. An on-site system for early infant diagnosis and ART initiation functioned better than a fragmented service spread across multiple sites. New emphasis on early diagnosis and rapid initiation of ART in the first weeks of life are essential to further reduce infant mortality.
